# Data-Mining Approach on Transcriptomics and Methylomics Placental Analysis Highlights Genes in Fetal Growth Restriction

**DOI:** 10.3389/fgene.2019.01292

**Published:** 2020-01-09

**Authors:** Floris Chabrun, Noémie Huetz, Xavier Dieu, Guillaume Rousseau, Guillaume Bouzillé, Juan Manuel Chao de la Barca, Vincent Procaccio, Guy Lenaers, Odile Blanchet, Guillaume Legendre, Delphine Mirebeau-Prunier, Marc Cuggia, Philippe Guardiola, Pascal Reynier, Geraldine Gascoin

**Affiliations:** ^1^ Département de Biochimie et Génétique, Centre Hospitalier Universitaire, Angers, France; ^2^ Unité Mixte de Recherche (UMR) MITOVASC, Équipe Mitolab, Centre National de la Recherche Scientifique (CNRS) 6015, Institut National de la Santé et de la Recherche Médicale (INSERM) U1083, Université d’Angers, Angers, France; ^3^ Réanimation et Médecine Néonatales, Centre Hospitalier Universitaire, Angers, France; ^4^ Laboratoire du Traitement de l’Image et du Signal, INSERM, UMR 1099, Université Rennes 1, Rennes, France; ^5^ Département d’Information médicale et dossiers médicaux, Centre Hospitalier Universitaire, Rennes, France; ^6^ Centre de Ressources Biologiques, Centre Hospitalier Universitaire, Angers, France; ^7^ Département de Gynécologie Obstétrique, Centre Hospitalier Universitaire, Angers, France; ^8^ Service de Génomique Onco-Hématologique, Centre Hospitalier Universitaire, Angers, France

**Keywords:** data mining, methylomics, intrauterine growth restriction, multi-omics, text-mining, transcriptomics

## Abstract

Intrauterine Growth Restriction (IUGR) affects 8% of newborns and increases morbidity and mortality for the offspring even during later stages of life. Single omics studies have evidenced epigenetic, genetic, and metabolic alterations in IUGR, but pathogenic mechanisms as a whole are not being fully understood. An in-depth strategy combining methylomics and transcriptomics analyses was performed on 36 placenta samples in a case-control study. Data-mining algorithms were used to combine the analysis of more than 1,200 genes found to be significantly expressed and/or methylated. We used an automated text-mining approach, using the bulk textual gene annotations of the discriminant genes. Machine learning models were then used to explore the phenotypic subgroups (premature birth, birth weight, and head circumference) associated with IUGR. Gene annotation clustering highlighted the alteration of cell signaling and proliferation, cytoskeleton and cellular structures, oxidative stress, protein turnover, muscle development, energy, and lipid metabolism with insulin resistance. Machine learning models showed a high capacity for predicting the sub-phenotypes associated with IUGR, allowing a better description of the IUGR pathophysiology as well as key genes involved.

## Introduction

Intrauterine growth restriction (IUGR) is a frequent complication of pregnancy with a prevalence in up to 5% to 10% in the general population (Zhang et al., 2015). It is defined as a restriction of fetal growth during pregnancy, “a fetus that doesn’t reach its growth potential” (Vayssière et al., 2015). It can lead to a birth weight and/or length below the tenth percentile for a given gestational age in newborns, thus considered as “Small for Gestational Age” (Vayssière et al., 2015). IUGR represents a major public health problem, being one of the main causes of premature birth, perinatal mortality, and neurological and respiratory morbidities (Flamant and Gascoin, 2013). It is also suspected to be a determining factor in the development of cardiovascular diseases, obesity, and type 2 diabetes in adulthood (Gascoin and Flamant, 2013).

Fetal growth is a complex process that involves fetal genetics, nutrient and oxygen availability, and maternal nutrition, as well as growth factors and hormones from maternal, fetal, and placental origin (Murki, 2014). Fetal growth is inseparable from placental growth and requires a continuous supply of nutrients that is adapted to each period of pregnancy (Sharma et al., 2016).

IUGR remains a complex problem for the clinician. Placental dysfunction and vascular underperfusion are involved in the largest proportion of cases (Kaplan, 2007; Malhotra et al., 2019). It results from utero-placental insufficiency due to abnormal uterine artery remodeling in the first trimester of pregnancy and may or may not be associated with pre-eclampsia (PE). However, while many risk factors have been identified, placental insufficiency is still unexplained in up to 60% of cases (Malhotra et al., 2019).

Epigenetics (Xiao et al., 2016) and gene expression (Buffat et al., 2007; Madeleneau et al., 2015) reprogramming play a central role in IUGR. However, the pathophysiological connections between these two fields of high-throughput analyses have only recently begun to be studied (Ding and Cui, 2017). Although many tools have been developed to analyze and integrate multi-omics data, this task remains a challenge in medicine (Gomez-Cabrero et al., 2014). Many features originating from the variance between samples and the complexity of the statistical data processing require developing data-driven approaches rather than classical hypothesis-driven approaches (van Helden, 2013). The exploration of pathophysiological conditions with such data-driven approaches must integrate many processes from clinical and biological data collection, through complex data normalization and mathematical and bioinformatics modeling, to the final interpretation and data visualization.

When dealing with a short list of genes, the exploration of their roles and underlying patterns is usually carried out through “manual” interpretation, using both annotations and personal knowledge. This “manual” interpretation may be used to categorize the genes, or to seek patterns in roles, functions, or localizations, underpinning the pathology or context studied. When dealing with thousands of significant gene features (e.g. expression levels or methylation levels), the interpretation becomes humanly untenable, due to time and memory limits. Rather than limiting our literature review to a small subset of the most significantly altered genes, we used text-mining algorithms to perform an unsupervised analysis of those genes. Those algorithms have already been used to categorize and summarize text corpora based on similarities in their content (Aggarwal and Zhai, 2012).

With the aim of having an extended vision of the pathophysiological processes at the origin of IUGR, while identifying the most predominant deregulated pathways that may be targeted for therapeutic purposes, we used machine-learning models to explore the relationship between placental transcriptomics and methylomics variations and IUGR. The highly predictive models obtained from IUGR and its sub-phenotypes were then used to highlight the genes with a high correlation with IUGR clinical severity, and thus with a high therapeutic potential.

## Material and Methods

**Figure 1:**
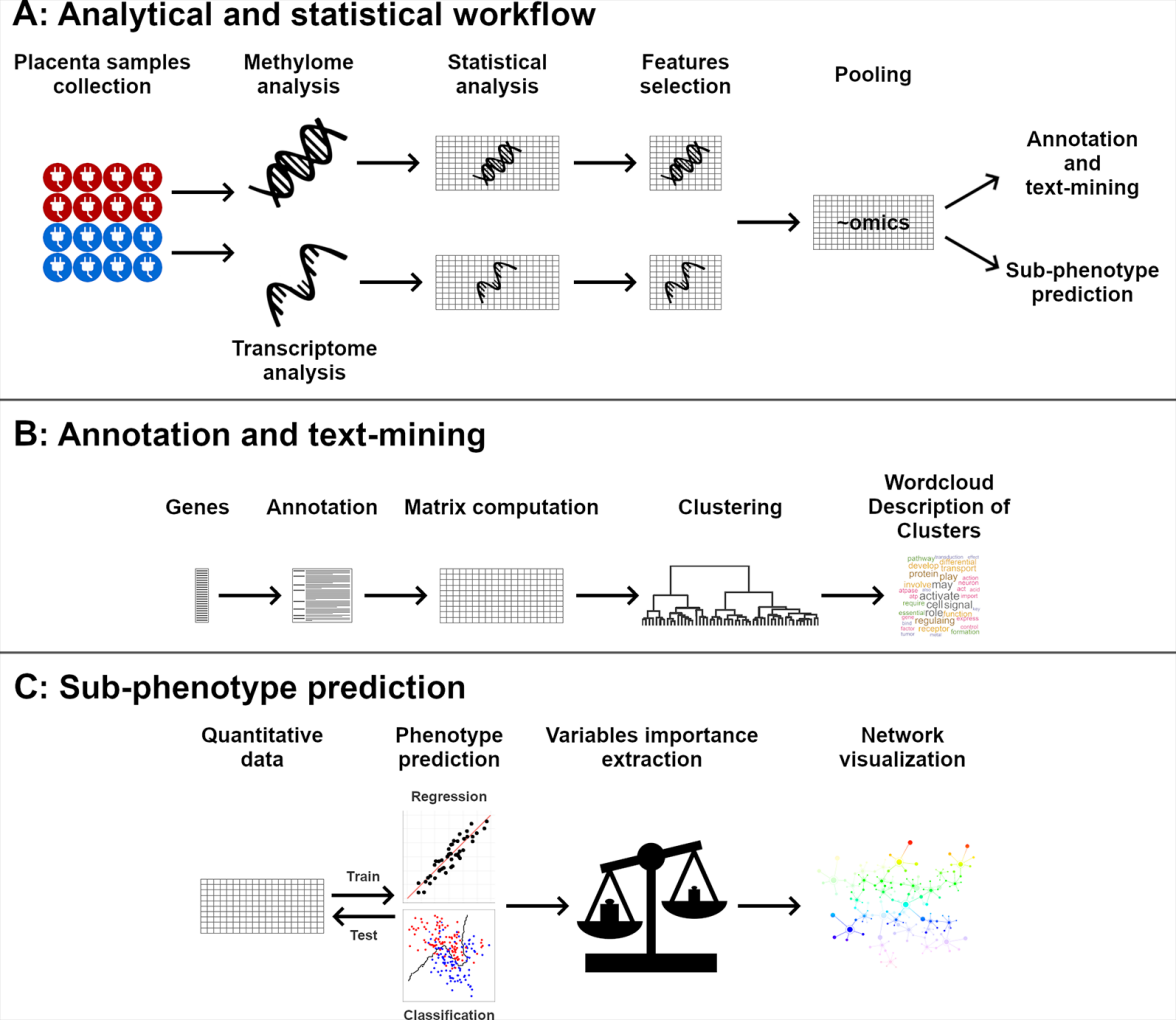
Global workflow of the analysis. Placentas methylome and transcriptome were analyzed **(A)**. Significant genes were clustered and described using text annotations **(B)**. Quantitative data were used to predict phenotypic data, and the importance of each gene in phenotype prediction was visualized using networks **(C)**.

The global workflow is summarized in **Figure 1**.

### Patients

All placentas were collected from Angers University Hospital. This study was approved by the Ethics Committee of Angers. All patients gave their informed consent for the use of their placenta. Clinical data related to the mother and the fetus, as well as neonatal data, were collected from the patients’ obstetric files. The cohort was registered at the French *CNIL* (*Commission Nationale de l’Informatique et des Libertés* no. pWP03752UL, ethics committee for the collection of clinical data from patient records). The study was validated by the French *CPP* (*Comité de Protection des Personnes*) and registered to the French Ministry of Research under number DC-2011-1467. The study was conducted in accordance with the declaration of Helsinki.

Placentas were obtained from caesarean sections before onset of labor or from vaginal delivery. For the analysis, patients were classified into two groups: IUGR and control group. The IUGR group was defined by a reduction of fetal growth during gestation, with a notch observed by Echo-Doppler in at least one uterine artery and with Doppler abnormalities on umbilical Doppler and/or cerebral Doppler and/or ductus venosus, and with a birth weight below the tenth percentile according to Audipog growth curves (American College of Obstetricians and Gynecologists, 2013) and confirmed by the anatomopathological analysis of the placenta after birth. The control group was defined by women with normal pregnancy and who underwent a planned caesarean section. All obstetrical and neonatal data were collected prospectively from medical records.

### Placental Samples

To avoid degradation, only placental tissues dissected within a time frame of 30 min after delivery were included. After removal of the maternal decidua and amniotic membrane, sections of 1 cm^3^ of placental villi were dissected from four different cotyledons between the basal and chorionic plates, as previously described (Gascoin-Lachambre et al., 2010). After vigorous washing with PBS to remove maternal blood, tissues were immediately frozen in liquid nitrogen, before storage at −80 °C, to further extract DNA and RNA. Placentas were then sent for anatomopathological analysis or stored at the biological core facility at Angers University Hospital.

### DNA Preparation and Microarray Hybridization

Genomic DNA extraction was performed manually using a QIAamp DNA mini QIAcube Kit (Qiagen, Venlo, Netherlands), according to the manufacturer’s protocol.

DNA was treated with bisulfite using an EZ-96 DNA Methylation Kit on a Zymo Spin I-96 column (Zymo Research, Irvine, CA, U.S.A.). Bisulfite-converted DNA was amplified, fragmented, and hybridized to Illumina Human Methylation 450k microarrays using an Illumina Hybridization Oven (Illumina, San Diego, CA, U.S.A.), according to the manufacturer’s protocol. Slides were analyzed by an Illumina I-Scan (Illumina, San Diego, CA, U.S.A.).

Raw iDAT files were directly imported in R software (R Development Core Team, 2008) and processed using the R minfi package (Aryee et al., 2014). Raw data were normalized using functional normalization (Fortin et al., 2014) before constructing the beta matrix for all 36 samples and 485,512 CpG sites (methylomics dataset).

### RNA Preparation and Microarray Hybridization

Total RNA was extracted after lysing samples with TRIzol reagent (Life Technologies, Carlsbad, CA, U.S.A.), using the RNeasy Micro kit (Qiagen, Venlo, Netherlands), according to the manufacturer’s recommendations. Biotinylated, amplified cRNA was generated using the Illumina Total Prep RNA Amplification kit (Ambion, Life Technologies, Carlsbad, CA, U.S.A.), according to the manufacturer’s recommendations. cRNA was hybridized on Illumina HumanHT-12 v4 Expression BeadChips, stained, and detected with the iScan system, according to the manufacturer’s protocol (Illumina, San Diego, CA, U.S.A.). A total of 47,323 marker probes were assessed, of which: 47,231 elements with sequences, with 46,841 with at least one genome alignment, including 34,627 elements mapped to at least one among 22,283 unique genes. GenomeStudio 2011 (version 1) and its Expression Analysis Module (version 1.9.0) were used for signal extraction and quantile normalization (Illumina, San Diego, CA, U.S.A.).

Normalized data for all 47,323 marker probes and 36 samples were imported into R software (R Development Core Team, 2008) and processed as described below (transcriptomics dataset).

### Omics Data Integration

Each omics dataset was processed independently. Levene’s tests were used to assess the comparability of variances between control and IUGR groups. Significant features were determined using Student’s t-tests. Alpha thresholds for *p*-value significance were set to α = 0.05. For Student’s t-tests, *p*-values were adjusted into *q*-values using the Benjamini-Hochberg method in order to control the false discovery rate. The $\frac{IUGR}{control}$ fold-change was computed for all significant features. Only features with Levene’s test *p*-value ≥ 0.05 and Benjamini-Hochberg adjusted Student’s t-test q-value < 0.05 were considered significant.

### Gene Annotation and Text-Mining

All genes showing a significant alteration in methylation or expression were annotated using abstracts available on PubMed, by automatic retrieval. Genes without available annotations were discarded. Abstracts were pre-processed by removing punctuation, short words (words of three characters or fewer) and stop words (i.e. common language non-specific words), and stemming (Willett, 2006). They were then analyzed by taking into account, in the same analytical process, unigrams, bigrams, and trigrams, commonly denoted as terms. A normalized term-frequency inverse-document-frequency (tf-idf) matrix (Aggarwal and Zhai, 2012) was then computed based on the frequency and specificity of each term in each gene summary, using the formula:

$$M_{i,j}=tf_{i}\times idf_{i}$$

With the inverse document frequency *idf*_i_ for the term *i*:

$$idf_{i}=\log_{2}\left(\frac{\left|D\right|}{\left|\{d|t_{i}\in d|\}\right|}\right)$$

where *Mi,j* is the value in the matrix for the term *i* and gene *j*,*tf_i_* is the number of occurrences of the term *i* in the gene *j* summary divided by the total number of terms in the summary, |*D*| is the number of genes and |{*d|t_i_* ∈ d|}| is the number of gene summaries where the term *i* appears.

Due to the large dimension of the initial tf-idf matrix, a Latent Semantic Analysis (LSA) (Evangelopoulos, 2013) was performed in order to reduce its dimension and render further analyses possible. K-means was then used to perform clustering based on gene annotations similarity. Clusters were then summarized by terms closest to the cluster centers.

### Phenotype Prediction and Network Visualization

Support vector machines (SVM) are state-of-the-art machine-learning models that have already been successfully applied to several omics studies (Ben-Hur et al., 2008). They can successfully highlight non-linear correlations between genes and phenotypic traits, in order to highlight genes based on their links with several phenotypic traits (Altmann et al., 2010). Furthermore, SVM models are particularly suitable for high-dimensionality datasets, such as results of high-throughput analyses (Vanitha et al., 2015).

SVM models were trained using grid search cross-validation to predict four phenotypic traits as a function of omics data: control/IUGR group, premature birth (see below), birth weight, and head circumference at birth. These four phenotypic traits were chosen because of their known relevance in the IUGR pathophysiology. Term birth is defined by the International Classification of Diseases as between 37 (included) and 42 (excluded) weeks (Quinn et al., 2016), otherwise 39.43 ± 2.43 weeks. To simplify, pregnancy term was expressed as a variable named premature birth, computed with the formula:

Premature birth=39−Gestational Age

Since gestational age and the newly-created variable, premature birth, are linearly correlated, this simplifies yet does not alter the interpretation of the results of the model’s predictions. Values >2 therefore indicate pre-term newborns, while values ≤-3 indicate post-term newborns.

Both head circumference at birth and birth weight were expressed as Z-scores according to the gestational age and gender, based on Olsen growth curves (Olsen et al., 2010), to standardize values between infants born at different terms. Case-control classification is important to verify the integrity of the dimension-reduced dataset. Birth weight is a criterion of severity of the IUGR. Head circumference at birth is a criterion of high severity, due to the brain sparing effect (Cohen et al., 2015). Premature birth is indirectly linked to severity of these. Indeed, in most cases during IUGR pregnancies, a delivery is induced or carried out *via* caesarean section, to prevent either maternal or fetal damage. Exploring factors correlated with the premature birth may therefore allow exploring severity symptoms not directly and only linked to IUGR.

The dimensionality of the omics dataset had to be reduced before training the SVM, to reduce noise and achieve better model predictions (Keogh and Mueen, 2010). For this reason, only features with a significant difference between IUGR and control groups were used to train SVM models (*q* < 0.05, after Benjamini-Hochberg adjustment). Several methods may be used to reduce the dimensionality of a dataset (Guyon and Elisseeff, 2003). Features selection was preferred compared to other methods like Principal Components Analysis as it allows the use of the initial variables instead of computing new, abstract dimensions, making the final interpretation easier. Student’s t-tests have already been evidenced as an effective method for features selection (Haury et al., 2011). By using Student’s t-tests as the features selection method, this step could be applied seamlessly to our omics analyses results, without modifying or altering the results.

The dataset was randomly partitioned into training and test sets, with a ratio of two-thirds/one-third, using stratified sampling in order to respect the original $\frac{case}{control}$ ratio. Due to the low number of samples and the imbalance between IUGR and control samples, Synthetic Minority Over-sampling Technique (SMOTE) was used in order to synthetically increase the training set size (Chawla et al., 2002). Test sets were not modified to ensure unbiased results when measuring models’ performances. Hyperparameters were fine-tuned with grid search cross-validation. Model results were assessed using accuracy for classification, and Pearson’s correlation factor for regression.

The variable importance for predicting each phenotypic trait was computed for each feature by Permutation Importance (Breiman, 2001). These results were used to carry out a network visualization to assess the importance of each feature in the prediction of each phenotypic trait.

### Computational Tools

R software (version 3.4.1) and Python (version 3.6) were used to carry out all data processing and analysis, as well as to output all plots (van Rossum, 1995; R Development Core Team, 2008). Heat maps were created using the gplots package (Warnes et al., 2016). Gene functional annotation analysis was performed for both gene expression and gene methylation using the DAVID 6.8 online tool (Huang et al., 2009a, 2009b). Genes were annotated with abstracts available from PubMed (10/10/2019) using easyPubMed (Fantini, 2019). Text-mining and SVM computing were processed using the python scikit-learn library (Pedregosa et al., 2011). Word clouds were created using the wordcloud R software package (Fellows, 2014). Hierarchical clustering was performed using the R software base package. Networks were constructed using Cytoscape (Shannon et al., 2003). The GIMP software was used to refine figures.

## Results

### Cohort

Patient cohort is described in **Table 1**. It should be noted that while the control group is smaller, controls are much more homogeneous concerning clinically relevant phenotypic traits discussed below. F-tests show a significantly lower variance in this control group for gestational age at birth (in grams) (*p* = 4.48E-5), head circumference at birth (in centimeters) (*p* = 1.08E-3), and APGAR at 5 min (*p* = 3.48E-5).

**Table 1:** Description of the patient cohort. *p*-values were computed using Wilcoxon tests (quantitative values) or Fisher tests (percentages).

			Control group (n = 8)		IUGR group (n = 28)		p
**Maternal data**	Age (years)		35.4 ± 3.9	8	29.1 ± 5.9	28	0.006
	BMI before pregnancy	(kg/m^2^)	23.7 ± 7.0	8	25.1 ± 7.9	28	N.S.
	Tobacco consumption	Before pregnancy	0 (0.0%)	8	2 (7.1%)	28	N.S.
		During pregnancy	0 (0.0%)	8	9 (32.1%)	28	N.S.
	Ethnic group	European	7 (87.5%)	8	26 (92.9%)	28	N.S.
		North African	1 (12.5%)	8	2 (7.1%)	28	N.S.
**Obstetric data**	Gestity		4.0 ± 2.1	8	2.5 ± 1.9	28	0.03
	Parity		2.6 ± 1.3	8	1.4 ± 0.9	28	0.005
	Weight gain (kg)		10.5 ± 10.5	8	9.1 ± 6.4	24	N.S.
	Type of delivery	Vaginal delivery	0 (0%)	8	5 (17.9%)	28	N.S.
		C-section	8 (100%)	8	23 (82.1%)	28	N.S.
	Pathology	IUGR	0 (0%)	8	16 (57.1%)	28	N/A
		IUGR + PE	0 (0%)	8	12 (42.9%)	28	N/A
**Newborn data**	Gestational age (week)		38.7 ± 0.7	8	34.0 ± 3.9	28	<0.001
	Gender	Boy	4 (50.0%)	8	9 (32.1%)	28	N.S.
		Girl	4 (50.0%)	8	19 (67.9%)	28	N.S.
	Birth weight	(Z-score)	−0.07 ± 0.89	8	−2.02 ± 0.75	28	<0.001
		(g)	3346 ± 444	8	1,524 ± 664	28	<0.001
	Birth size	(Z-score)	−0.47 ± 0.74	7	−1.90 ± 0.80	26	<0.001
	Birth size	(cm)	49.2 ± 1.8	7	39.2 ± 5.2	26	<0.001
	Head circumference at birth	(Z-score)	0.22 ± 0.49	7	−1.30 ± 0.86	27	<0.001
	Head circumference at birth	(cm)	34.6 ± 0.9	7	29.0 ± 3.4	27	<0.001
	APGAR at 5 min		9.88 ± 0.35	8	9.11 ± 2.08	28	N.S.
	Resuscitation at birth		0 (0%)	8	12 (42.9%)	28	0.03
	NICU		0 (0%)	8	18 (64.3%)	28	0.003

BMI, body mass index; PE, pre-eclampsia; NICU, neonatal intensive care unit; N.S., non-significant versus α = 0.05; N/A, not applicable.

### Univariate Analyses

A total of 1651 features (1,072 DNA methylation sites, 579 transcripts) showed significantly different values between IUGR and control groups (*q* < 0.05). The full list of significant features is available in **Supplementary Table 1**.

Since a significant difference in mean gestational age had been observed between IUGR and control groups, univariate analyses were re-run after excluding IUGR samples with a gestational age lower than 37 weeks. Kendall correlation tests were then performed to compare Student’s t-tests results obtained for the whole cohort and for the high gestational age restricted subset. Gene expression and gene methylation features were significantly correlated (*p* < 0.001, τ = 0.45; *p* < 0.001, τ = 0.40, respectively).

**Figure 2:**
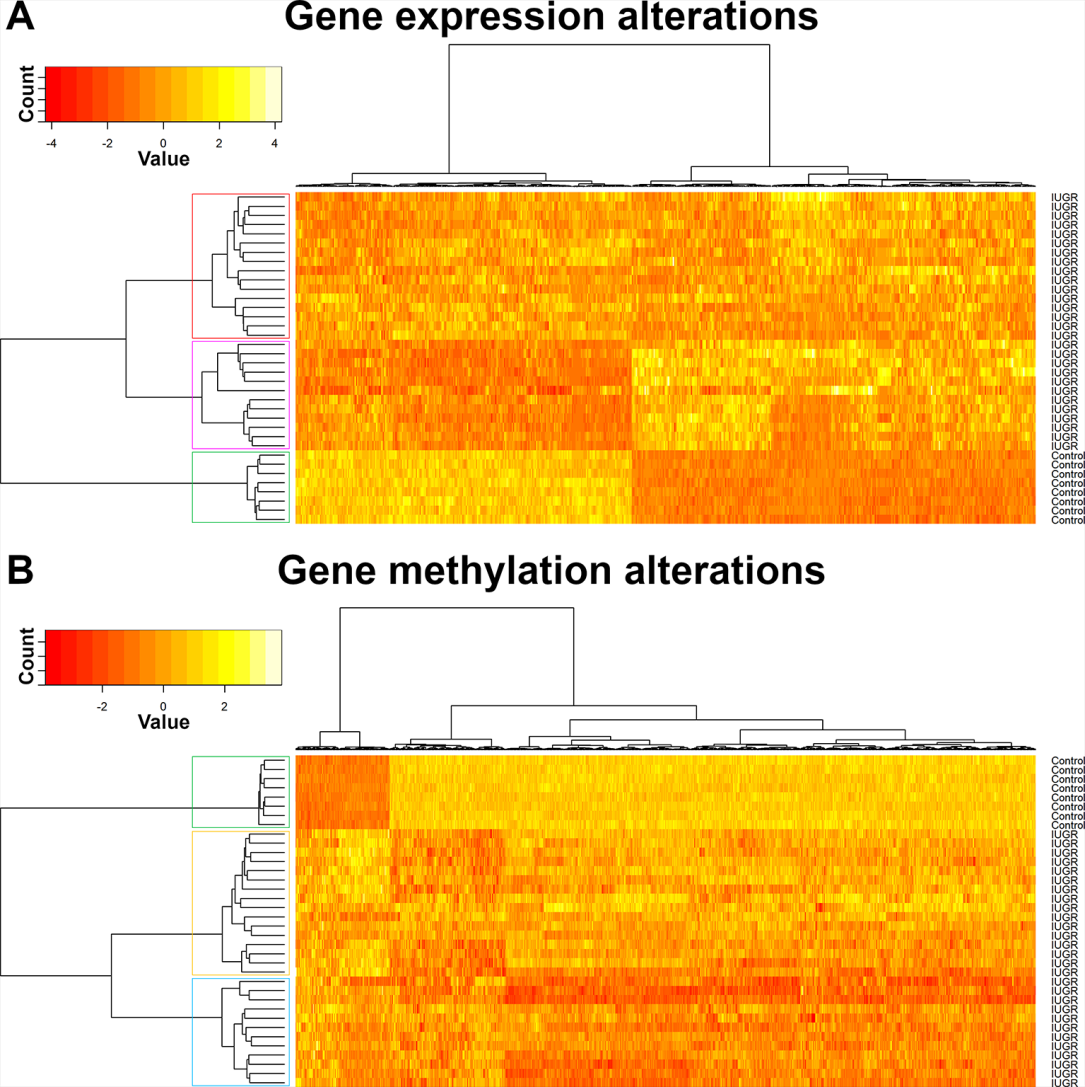
Hierarchical clustering of samples, gene expression **(A)** and methylation **(B)**.

**Figure 3:**
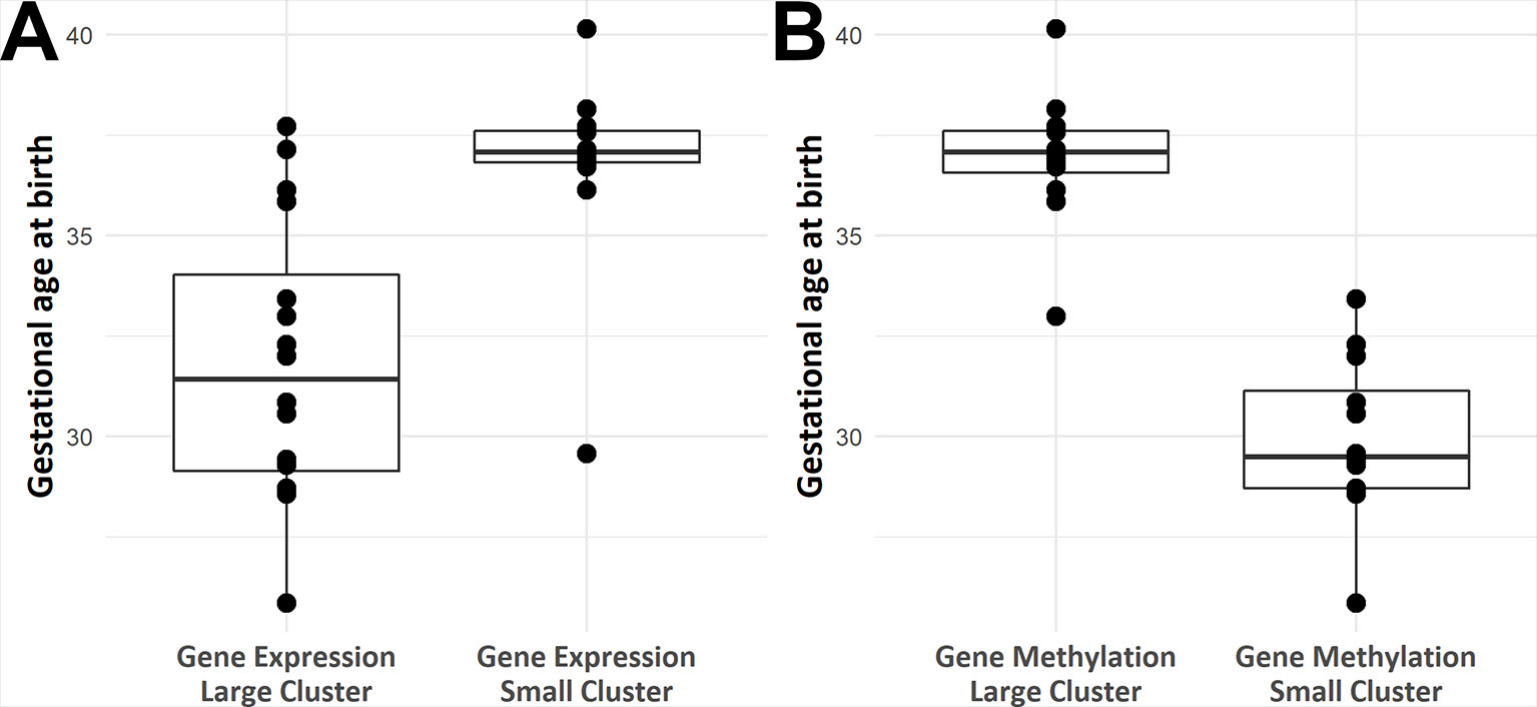
Box plots of gestational age at birth according to IUGR samples position in hierarchical clustering based on methylomics **(A)** and transcriptomics **(B)** data.

Heat maps picturing all genes with significant expression (**Figure 2**) or methylation (**Figure 2**) alteration showed a global hypomethylation, as opposed to a balanced ratio between the number of overexpressed and underexpressed transcripts. While hierarchical clustering distinctly separated IUGR from control samples, IUGR samples appeared divided into two different clusters for both heatmaps, even though the exact distribution of IUGR samples is not exactly the same for epigenetic and expression alterations. In order to explain this behavior, gestational age at birth of IUGR samples according to clusters was plotted in **Figure 3**.

Gene functional annotation analysis, performed with DAVID, showed gene expression and/or methylation alterations significantly associated with several pathways (*p* < 0.05), including: NAD-binding, histone acetylation, mTOR signaling pathway, lysosome, cell-cell adhesion and cell junction, calmodulin binding, and carbohydrates metabolism. The complete results are available in **Supplementary Table 2**.

Only 25 genes were found to be altered both in methylome and transcriptome (**Table 2**). Among these 25 genes, eight show a significant linear correlation between methylation and expression.

**Table 2:** Genes found altered in both methylome and transcriptome. Numbers in brackets refer to the number of methylation sites (methylome) and transcripts (transcriptome) found significantly altered.

Gene symbol	Gene name	Epigenetics (sites count/total)	Gene expression (transcripts count/total)	r
***PAPPA2***	*Pregnancy-Associated Plasma Preproprotein-A2*	Hypomethylated (2/13)	Overexpressed (2/2)	-0.76
***AP2A1***	*Adaptor Related Protein Complex 2 Subunit Alpha 1*	Hypomethylated (1/26)	Underexpressed (2/3)	N.S.
***BCL6***	*B Cell CLL/Lymphoma 6*	Hypomethylated (2/55)	Overexpressed (1/2)	-0.65
***SLC2A1***	*Solute Carrier Family 2 Member 1*	Hypomethylated (2/34)	Overexpressed (1/1)	-0.42
***UNKL***	*Unkempt Family Like Zinc Finger*	Hypomethylated (2/74)	Underexpressed (1/3)	N.S.
***WSB1***	*WD Repeat and SOCS Box Containing 1*	Hypomethylated (1/19)	Underexpressed (2/3)	N.S.
***AFAP1***	*Actin Filament Associated Protein 1*	Hypomethylated (1/103)	Overexpressed (1/3)	N.S.
***ALDOA***	*Aldolase, Fructose-Bisphosphate A*	Hypomethylated (1/27)	Overexpressed (1/4)	-0.43
***ALKBH5***	*AlkB Homolog 5, RNA Demethylase*	Hypomethylated (1/23)	Overexpressed (1/1)	N.S.
***C1QTNF1***	*C1q And TNF Related 1*	Hypomethylated (1/40)	Underexpressed (1/3)	0.40
***CALM1***	*Calmodulin 1*	Hypermethylated (1/20)	Overexpressed (1/1)	N.S.
***DGKZ***	*Diacylglycerol Kinase Zeta*	Hypomethylated (1/62)	Overexpressed (1/3)	N.S.
***DLX5***	*Distal-Less Homeobox 5*	Hypomethylated (1/47)	Overexpressed (1/1)	N.S.
***FLNB***	*Filamin B*	Hypomethylated (1/40)	Overexpressed (1/1)	-0.58
***FOXK1***	*Forkhead Box K1*	Hypomethylated (1/175)	Underexpressed (1/2)	0.36
***LIMCH1***	*LIM and Calponin Homology Domains 1*	Hypomethylated (1/51)	Overexpressed (1/1)	-0.51
***PDP2***	*Pyruvate Dehyrogenase Phosphatase Catalytic Subunit 2*	Hypomethylated (1/13)	Underexpressed (1/2)	N.S.
***PDXK***	*Pyridoxal Kinase*	Hypomethylated (1/37)	Underexpressed (1/1)	N.S.
***PEA15***	*Proliferation and Apoptosis Adaptor Protein 15*	Hypomethylated (1/12)	Overexpressed (1/1)	N.S.
***PLEKHA2***	*Pleckstrin Homology Domain Containing A2*	Hypermethylated (1/22)	Overexpressed (1/4)	N.S.
***RALGPS1***	*Ral GEF With PH Domain and SH3 Binding Motif 1*	Hypomethylated (1/20)	Underexpressed (1/1)	N.S.
***RRAD***	*RRAD, Ras Related Glycolysis Inhibitor and Calcium Channel Regulator*	Hypomethylated (1/13)	Overexpressed (1/2)	N.S.
***SFRS8***	*Splicing Factor SWAP*	Hypomethylated (1/77)	Underexpressed (1/1)	N.S.
***UCKL1***	*Uridine-Cytidine Kinase 1 Like 1*	Hypomethylated (1/18)	Underexpressed (1/1)	N.S.
***USP5***	*Ubiquitin Specific Peptidase 5*	Hypomethylated (1/23)	Underexpressed (1/1)	N.S.

Pearson’s correlation coefficient *r* is given for genes with a significant correlation between methylation and expression. N.S., Not significant.

### Textual Annotation and Text-Mining

Among these 1,651 features, 1,269 unique genes could be identified, and textual annotations were successfully retrieved for 1,259 of them. A total of 196,918 abstracts were retrieved (95% confidence interval: [146;167] abstracts per gene). LSA allowed reducing the dimension from 135,220 unique terms among all abstracts to 1,000 principal components, while retaining 97% of the initial tf-idf matrix variance. Genes were classified into 24 clusters. The cluster sizes ranged from 7 (0.6%) to 241 (19.1%) genes.

**Figure 4:**
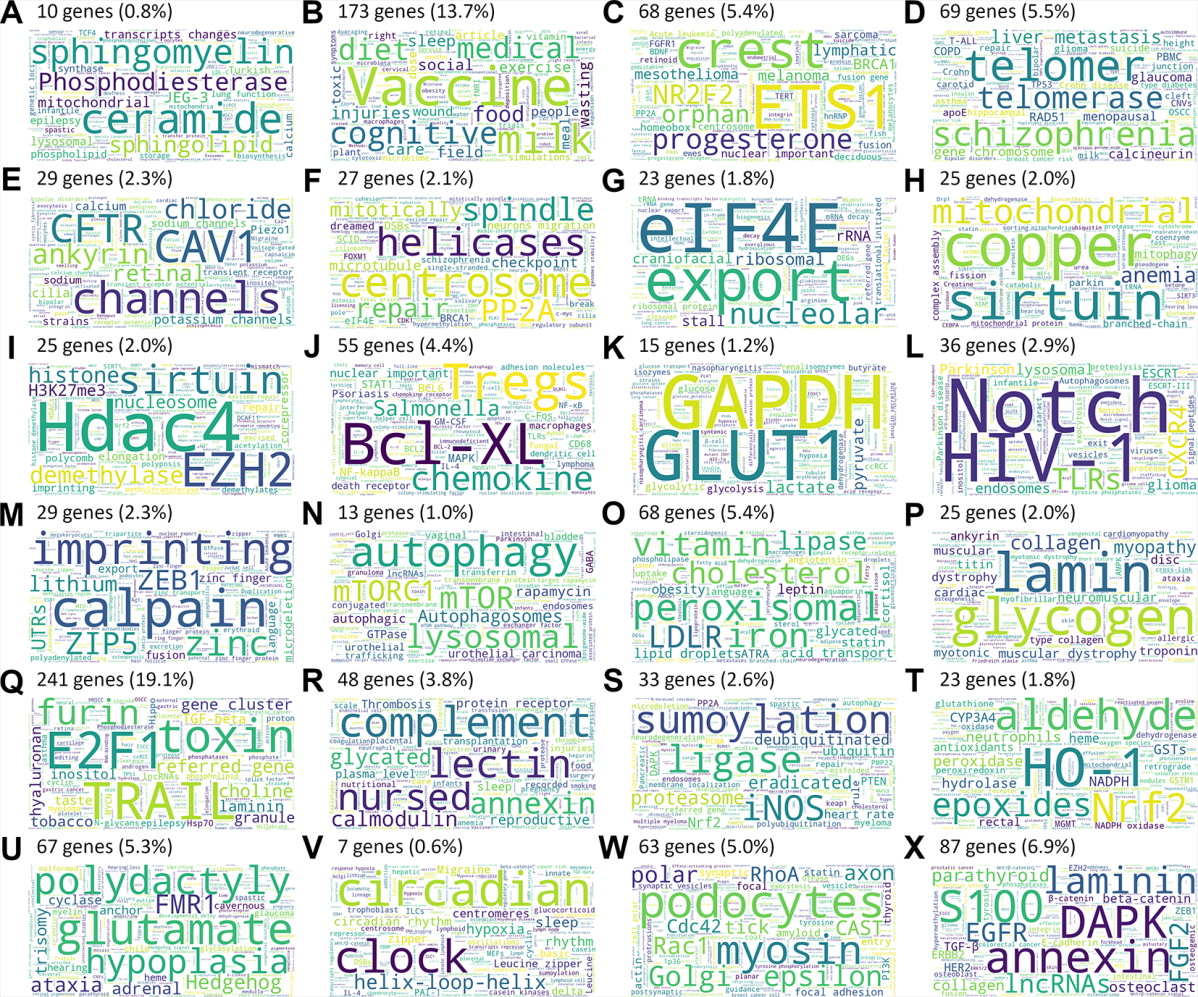
Word clouds summarizing the most frequent and specific terms among the 24 gene clusters **(A–X)**.

These clusters were summarized by word clouds picturing the most frequent and specific terms among the gene clusters, allowing a quick and easy grasp and visualization of the global role of the clusters (**Figure 4**).

### Predicting Phenotypic Traits From Omics Data

**Figure 5:**
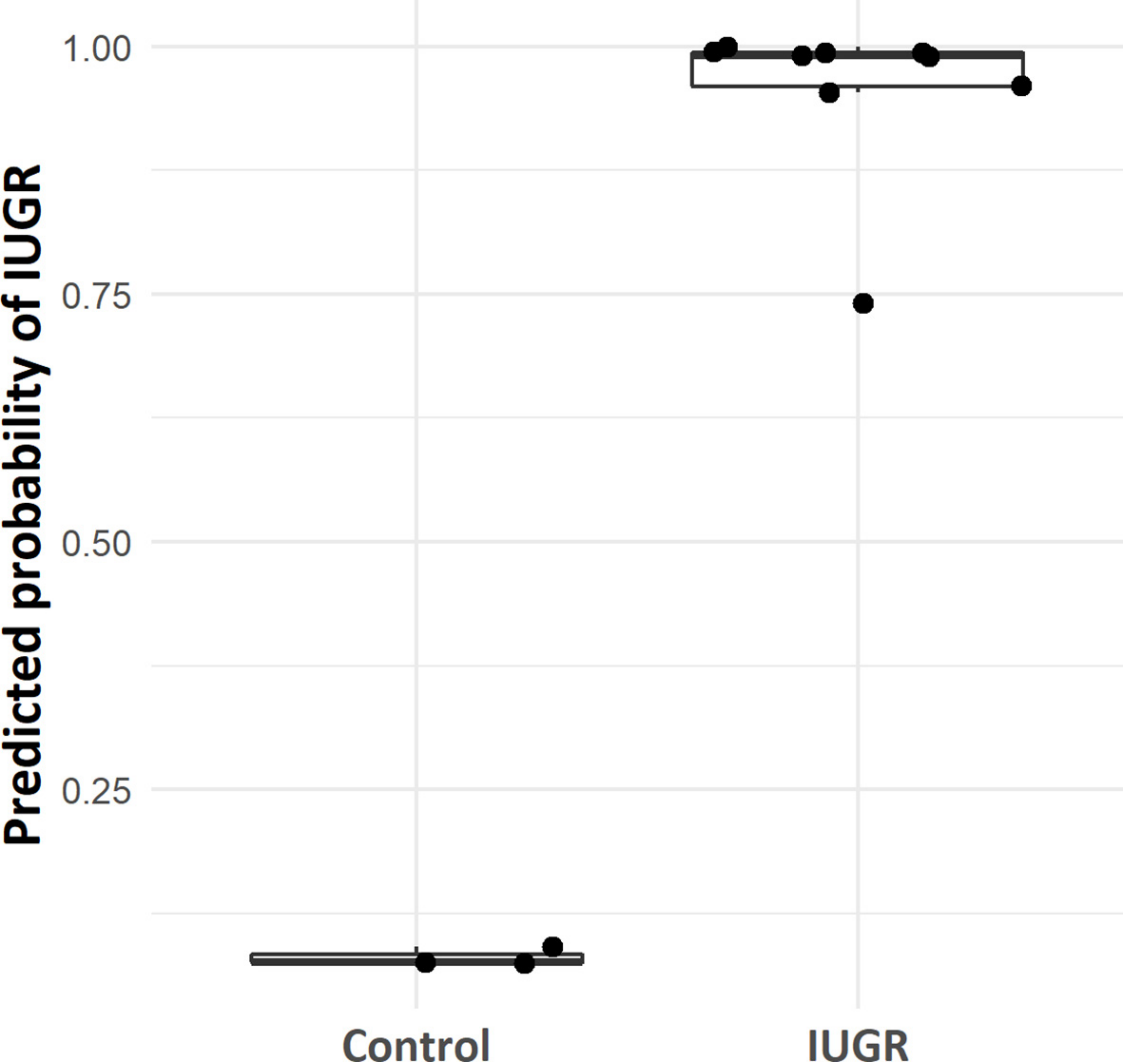
Box plot of case-control model predicted probability according to IUGR/control group.

The 1,651 features were used as input data to predict the outcome for four phenotypic traits (IUGR, premature birth, birth weight, and head circumference), in order to measure the importance of each gene in sub-phenotypic prediction. Class-control classification showed perfect predictions on the test set, with clearly distinct predicted probabilities between control and IUGR samples (**Figure 5**). This large gap of probabilities between IUGR and control samples confirmed the robustness of the model. These results were expected, as only features showing a significant difference between IUGR and control groups were selected for training the model. Furthermore, the previous unsupervised analysis (**Figure 2**) confirmed a clear distinction between IUGR and control samples.

**Figure 6:**
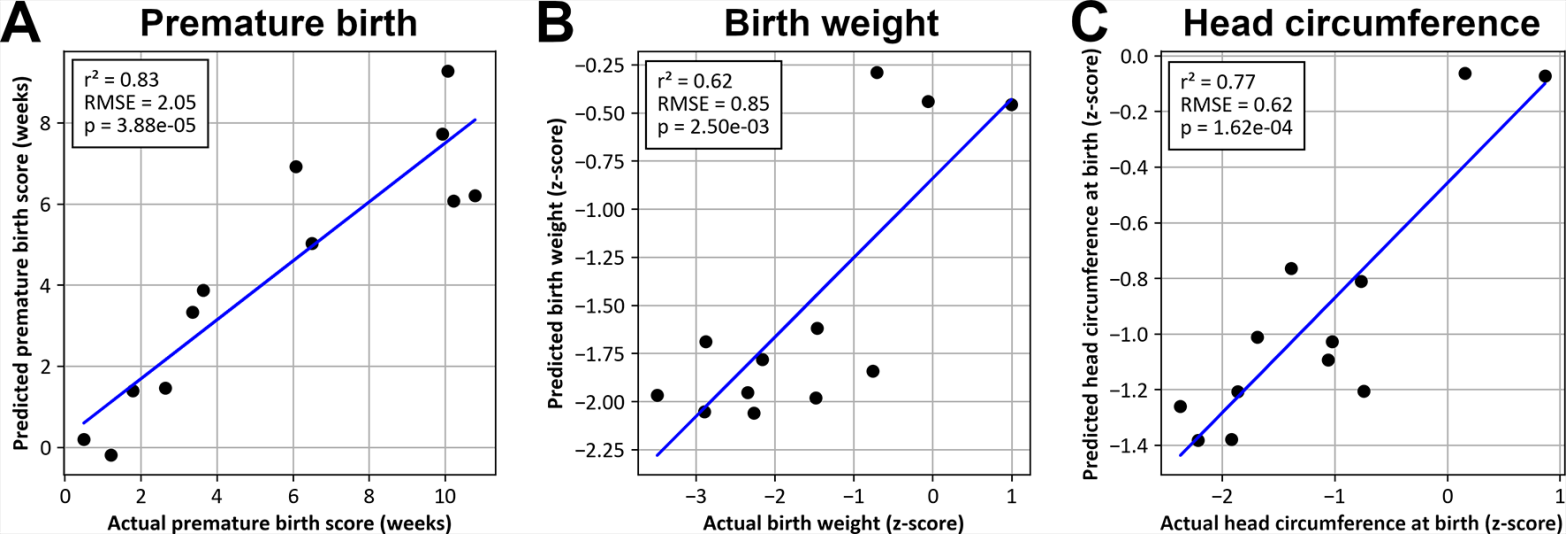
Values predicted by SVM models as a function of actual values for premature birth **(A)**, birth weight **(B)**, and head circumference at birth **(C)**.

Premature birth, birth weight, and head circumference scores predicted on test samples were linearly correlated with actual values (*p* < 0.01) (**Figure 6**).

**Figure 7:**
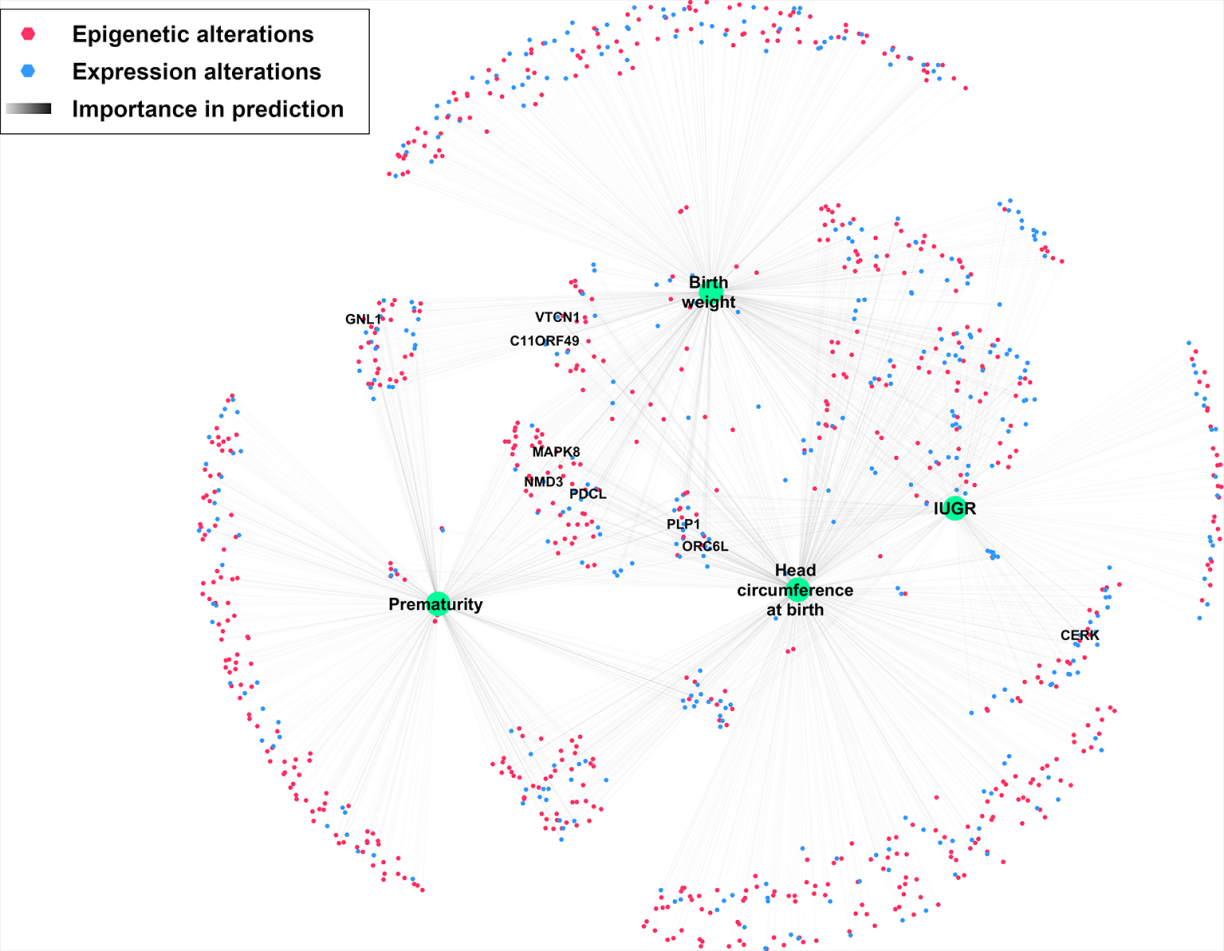
Network depicting significantly altered features and their importance in predicting IUGR phenotype. Nodes were positioned according to an Edge-weighted Spring Embedded Layout, based on feature importance for predicting each phenotypic trait. Only genes with at least 80% importance for predicting at least one phenotypic trait are labeled.

A network was created to represent all omics features with at least 10% importance for predicting at least one phenotypic trait (**Figure 7**). Among the nine genes with high importance (> 80%) in the prediction of at least one phenotypic trait, five (NMD3, ORC6L, MAPK8, PDCL, PLP1), in the center of the network share an importance in predicting most studied phenotypic traits.

The full list of methylomics and transcriptomics features with importance higher than 50% for phenotypic prediction is available in the **Supplementary Table 3**.

## Discussion

### Text Annotation Clustering and Word Cloud Visualization

In most high-throughput gene studies, functional annotation analysis is a powerful tool, allowing the highlighting of pathways enriched in a particular pathophysiological context. However, limiting gene annotation to categorical roles or pathways leads to a significant loss of knowledge in comparison with data available in literature.

Word clouds allowed a visual description of the main biological processes and pathways involved in the IUGR pathophysiology, in order to speed up and deepen the bibliographic work on genes significantly altered in IUGR.

#### Cell Signaling and Proliferation

Many terms among the most frequent and specific refer to proto-oncogenes and cell proliferation and signaling and development mechanisms. This is confirmed by several genes isolated from both methylome and transcriptome (overexpression of *BCL6*, *CALM1*, *DLX5*, *PEA15*, *RRAD*, and underexpression of *FOXK1* and *UCKL1*).

#### DNA, RNA Regulation, Transcription, Translation

Many gene clusters (C, D, F, G, I, L, respectively 5.4%, 5.5%, 2.1%, 1.8%, 2.0% and 2.9% of genes) refer to DNA methylation and repair, regulation of transcription, and RNA splicing and translation. Epigenetic and gene expression alterations in IUGR have been evidenced here as well as in literature (Kawai et al., 2015).

#### Mitochondria and Oxidative Stress

Clusters H and T (2.0% and 1.8%, respectively) refer to mitochondria alterations, cell death and apoptosis, and redox reactions. Indeed, pregnancy increases ROS production and oxidative stress, causing damage to mitochondria and potentially leading to cell death, especially during pathological pregnancies like PE or IUGR (Myatt and Cui, 2004). These phenomena may have a role in the fetal programming of atherosclerosis (Leduc et al., 2010). *ALKBH5* (found hypomethylated, overexpressed) encodes a hypoxia-inducible factor playing a role in cell proliferation (Zhang et al., 2016).

#### Intra- and Extra-Cellular Matrix

Several clusters (E, F, W, respectively 2.3%, 2.1% and 5.0%) suggest primarily cytoskeleton and cell-cell junction alterations. Furthermore, cluster N (1.0%) refers to intra-cellular trafficking and cell mechanisms relying heavily on the cytoskeleton. Riquelme and her colleagues (Riquelme et al., 2011) have already evidenced abnormalities in the lipid raft composition of the microvillous membrane of the placental syncytiotrophoblast, linked with alterations in the expression of several cytoskeletal proteins (actin, ezrin, and cytokeratin-7) in placentas from pathological pregnancies (PE and IUGR). They suggest that these cytoskeleton alterations might be responsible for alterations in the syncytiotrophoblast microvilli, which may play a major role in the IUGR pathophysiology. Among the genes found altered in both methylome and transcriptome, *AFAP1* is a major regulator of the cytoskeleton structure (Xiao et al., 2012). *FLNB* codes for an actin-binding protein crosslinking actin filaments and playing various roles including cell proliferation and angiogenesis through mechanotransduction (Xu et al., 2017). Clusters P and X (2.0% and 6.9%, respectively) refer to extracellular matrix alterations. Such alterations have already been evidenced in IUGR (Merchant et al., 2004; Swierczewski et al., 2012).

#### Protein Degradation and Turnover

Cluster S (2.6%) refers to protein SUMOylation, ubiquitination, and degradation. It has been evidenced that protein ubiquitination is altered in IUGR and PE, particularly due to a modulation by oxidative stress, with an increased degradation of p53 and Mcl-1 proteins, contributing to the pathological mechanisms of the diseases (Rolfo et al., 2012). *WSB1* (underexpressed here) mediates ubiquitination and proteolytic degradation, and is also involved in cell and glucose metabolism, playing a role in hypoxia-related mechanisms (Haque et al., 2016). *USP5* (underexpressed here) codes for a deubiquitinating enzyme which has also been shown to play a role in cell cycle modulation.

#### Heart and Skeletal Muscle Development

Heart and skeletal muscles are referred to in cluster P (2.0%). Wang et al. (Wang et al., 2013) and Yates et al. (Yates et al., 2012) already reported that hypoxemia and hypoglycaemia undergone during IUGR decrease muscle mass in offspring. *DGKZ* (found hypomethylated, overexpressed) is known to induce muscle fiber hypertrophy and plays a role in the adaptation to energy metabolism alterations (Benziane et al., 2017). *FOXK1* induces muscle progenitor cell proliferation and inhibits their differentiation (Shi et al., 2012). *FOXK1* was found here both hypomethylated and underexpressed. This underexpression might be due to another role of *FOXK1* in repressing starvation-induced atrophy and autophagy (Bowman et al., 2014).

#### Energy Metabolism and Insulin Resistance

Major references are made to fat and lipid metabolism in cluster O (5.4%) and cluster Q (1.2%). These clusters support the hypothesis of an alteration of lipid and fat metabolism during IUGR, reflecting mechanisms of insulin resistance. Several genes found altered in both methylome and transcriptome support this pathway. Among these genes, *PAPPA-2* is the gene with the largest number of methylation sites significantly altered (hypomethylation), and with the largest number of transcripts significantly differently expressed (overexpression) in IUGR placentas. Its overexpression has already been reported in both maternal blood and the placenta in IUGR (Whitehead et al., 2013) and PE (Kramer et al., 2016). *PAPPA-2* encodes a protein cleaving the insulin-like growth factor 1 (IGF-1) from a ternary complex with IGF binding proteins (IGFBP-3) (Fujimoto et al., 2017). Via this regulation of the IGF-1 bioavailability, it plays a key role in both placenta development and fetal growth. Both low and high levels of IGF-1 have also been associated with insulin resistance (Friedrich et al., 2012). Interestingly, the *STC2* gene, encoding the PAPPA2 inhibitor stanniocalcin-2, was found significantly hypomethylated here, but its expression was not significantly altered between IUGR and control groups.

*PEA15* encodes a phosphoprotein responsible for insulin resistance and diabetes. Higher levels of expression of *PEA15* have been reported in both patients with diabetes mellitus type 2 (Condorelli et al., 1998) and in euglycemic patients with impaired insulin sensitivity (Valentino et al., 2006). The *DGKZ* gene, already discussed above, has been proven to play a role in the protection against peripheral insulin resistance and in improving overall energy metabolism (Benziane et al., 2017). *SLC2A1*, also known as glucose transporter 1 (*GLUT1*), is the major glucose transporter in the human placenta and the rate-limiting step of glucose transport from the placenta to the fetus (Illsley, 2000). Its overexpression here might reflect mechanisms of adaptation to fetal nutrient restriction. *C1QTNF1*, also known as glucose-dependent insulinotropic polypeptide (GIP) is an adipokine, whose secretion by adipocytes is increased under hypoxia, partially under the control of HIF-1α. It stimulates proinflammatory gene expression and impairs insulin sensitivity of adipocytes (Chen et al., 2015). However, *C1QTNF1* was found underexpressed in this study.

Two more genes supporting these mechanisms of insulin resistance were found here among the most overexpressed genes: *HTRA4* (IGF binding domain containing protein, fold-change = 7.33) and *LEP* (leptin, fold-change = 4.89). This major overexpression had already been observed in both IUGR (Madeleneau et al., 2015) and PE (Brew et al., 2016).

### Sub-Phenotype Prediction

Unsupervised clustering (**Figure 2**) showed a clear distinction between IUGR and controls and suggested the existence of multiple sub-phenotypes in the IUGR group (**Figure 3**).

As expected, SVM models were able to accurately predict such phenotypic traits: gestational age at birth, birth weight, and head circumference, using only a small subset of the whole data, i.e. 1,651 (0.3%) methylome and transcriptome variables. These results confirmed the high predictive value of the genes highlighted in this study in the IUGR, as well as in several variables of severity and pathophysiology of the IUGR.

In particular, nine genes with high importance in the prediction of these phenotypic traits were observed. Network visualization (**Figure 7**) showed that most of these genes are correlated with most clinically relevant traits studied here.

Among these genes, *CERK*, *GNL1*, *PLP1*, and *MAPK8* are known to be altered or play a direct role in the pathophysiology of IUGR or PE in various pathways discussed above: differentiation and proliferation regulation, response to hypoxia and oxidative stress, and neurological maturation (Vaiman et al., 2011; Reid et al., 2012; Goyal et al., 2013; Chan et al., 2019). For the other genes (*VTCN1*, *C11ORF49*, *PDCL*, *ORC6L*, *NMD3*), no obvious link with IUGR was found in literature, creating a topic for future studies regarding their exact role in the IUGR pathophysiology.

### Limits

Our study was mainly limited by the imbalance between cases and controls and the relatively weak number of controls. However, as already stated, controls show a significantly lower variance for most phenotypic traits discussed in this study. Furthermore, oversampling methods were used in order to compensate this limit and prevent model overfitting, while assessing the importance of genes on unmodified test sets which were not previously used for training models.

### Conclusion

Many epigenetic and gene expression alterations in IUGR placentas have been observed here, some of them confirming previous mechanisms already published, and others being new findings. Several major pathways were highlighted by annotation text-mining analysis: cell cycle and proliferation, regulation of apoptosis, epigenetic modifications, transcription, translation, oxidative stress and hypoxia, cytoskeleton and cell structure, protein degradation and turnover, autophagy, muscle development, and glucose and lipid energy metabolism. The involvement of these pathways was supported by significant differences in both methylome and transcriptome. Finally, several key genes with high correlation with phenotypic traits clinically relevant for IUGR were observed and may constitute potential targets for future study.

## Data Availability Statement

Array-based datasets for both genome methylation and expression have been deposited at the European Genome-phenome Archive (EGA), which is hosted by the EBI and the CRG, under accession number EGAS00001003467. Further information about EGA can be found on https://ega-archive.org (The European Genome-phenome Archive of human data consented for biomedical research, http://www.nature.com/ng/journal/v47/n7/full/ng.3312.html). Analysis output files are available in **Supplementary Material** (**Supplementary Tables 1**–**3**).

## Ethics Statement

The studies involving human participants were reviewed and approved by Ethics committee of Angers. The patients/participants provided their written informed consent to participate in this study.

## Author Contributions

FC: literature search, data analysis, data interpretation, figures, writing. NH: literature search, data collection, data analysis. XD: data interpretation. GR: data interpretation. GB proofreading, expertise in data analysis methods. JB: expertise in data analysis methods. VP: proofreading. GLen: proofreading. OB: data collection. GLeg: data collection. DM-P: proofreading. MC: proofreading, expertise in data analysis methods. PG: data analysis, data interpretation, expertise in data analysis methods. PR: co-director of the study, data interpretation, expertise, writing. GG: director of the study, literature search, data collection, data interpretation, expertise, writing.

## Funding

This study was funded by a grant from the Angers University Hospital, France.

## Conflict of Interest

The authors declare that the research was conducted in the absence of any commercial or financial relationships that could be construed as a potential conflict of interest.

## Abbreviations

IUGR, intrauterine growth restriction; PE, pre-eclampsia; Se, sensitivity; Sp, specificity; tf, term frequency; idf, inverse document frequency; SVM, support vector machine; RMSE, root-mean-square deviation; C-section, caesarean section.
